# Targeted *in vivo* delivery of EGFR siRNA inhibits ovarian cancer growth and enhances drug sensitivity

**DOI:** 10.1038/srep36518

**Published:** 2016-11-07

**Authors:** Minati Satpathy, Roman Mezencev, Lijuan Wang, John F. McDonald

**Affiliations:** 1 Integrated Cancer Research Center, 315 Ferst Drive, Atlanta, GA 30332, USA; 2 School of Biological Sciences, 315 Ferst Drive, Atlanta, GA 30332, USA; 3 Petit Institute for Bioengineering and BioScience, Georgia Institute of Technology, 315 Ferst Drive, Atlanta, GA 30332, USA.

## Abstract

A functionalized nanohydrogel siRNA delivery system and a mouse model of serous ovarian cancer were used to test predictions from previous cell line studies that knockdown of EGFR (epidermal growth factor receptor) may be of clinical significance in the treatment of epithelial tumors especially with respect to the enhancement of platinum based therapies. Our results support these predictions and suggest that targeted delivery of EGFR siRNA may be an effective strategy for the treatment of ovarian and other epithelial tumors associated with elevated levels of EGFR and especially those demonstrating resistance to platinum-based therapies.

Extensive genomic profiling of cancer tissues/cells has led to the identification of hundreds of putative gene “drivers” of the disease^1^. While the protein products of some of these genes are effectively inactivated by small inhibiting molecules, the vast majority (70–80%) of human genes are considered “non-druggable” on the protein level^2^. Additionally, since individual protein inhibitors are predicted to interact, on average, with >300 “off target” proteins^3^, the negative side effects associated with protein level drug therapy can be significant. For these reasons, there is increasing interest in the potential application of siRNAs and other small regulatory RNAs in the treatment of cancer^4^. However, a persistent hurdle to the clinical application of RNA therapy is the development of methodologies that can efficiently target delivery of therapeutic RNAs *in vivo*^5^. We report here on the successful use of functionalized nanohydrogels for the targeted delivery of siRNA against EGFR (epidermal growth factor receptor) in an ovarian cancer mouse model and the consequent inhibition of tumor growth and enhancement of cisplatin chemotherapy.

## Results and Discussion

We previously reported that core/shell nanogels composed of poly N-isopropylmethacrylamide (pNIPMAm) functionalized with an ephrin mimetic peptide (YSAYPDSVPMMS) to target the EphA2 receptor (ephrin type-A receptor 2), can effectively deliver siRNA against EGFR in ovarian cancer (OC) cells *in vitro*^6^. With the goal of testing the efficacy of this delivery system *in vivo*, we constructed a luciferase positive ovarian cancer cell line (Hey A8-F8) (Supplementary Fig. S1) that expresses elevated levels of both the EphA2 receptor and EGFR (Supplementary Fig. S2) and confirmed that EGFR levels are significantly reduced in these cells when targeted *in vitro* by our functionalized nanogel carrying EGFR siRNA (Supplementary Fig. S3).

**Figure 1:**
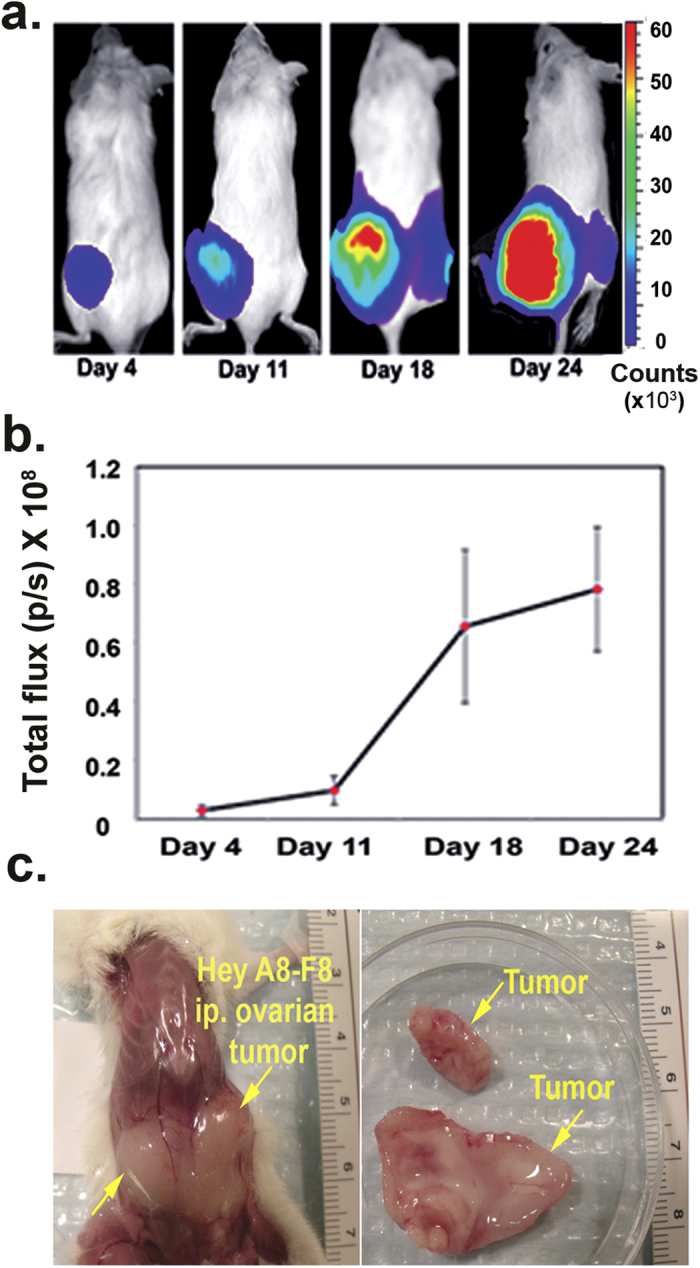
Bioluminescent imaging of *in vivo* tumor growth over time. (**a**) Images of the bioluminescence signal in representative mice at 4 time points after IP injection of Hey A8-F8 cells; (**b**) Quantitation of tumor growth expressed as average bioluminescence signal in a cohort of mice at 4 time points after IP injection of Hey A8-F8 cells. Each time point represents the mean (n = 12) level of total photon flux per second +/−SD; (**c)** Bright field image displaying tumor within the peritoneal cavity (left) in a whole animal and excised tumor tissues (right).

Hey A8-F8 cells implanted intra-peritoneal (IP) into NOD/SCID mice develop extensive cancer growth in the peritoneal cavity that can be noninvasively monitored by bioluminescence imaging (BLI) (Fig. 1a,b). The tumors grow as solid dense masses (Fig. 1c) with a histology characteristic of high-grade serous ovarian carcinomas (Supplementary Fig. S4).

**Figure 2:**
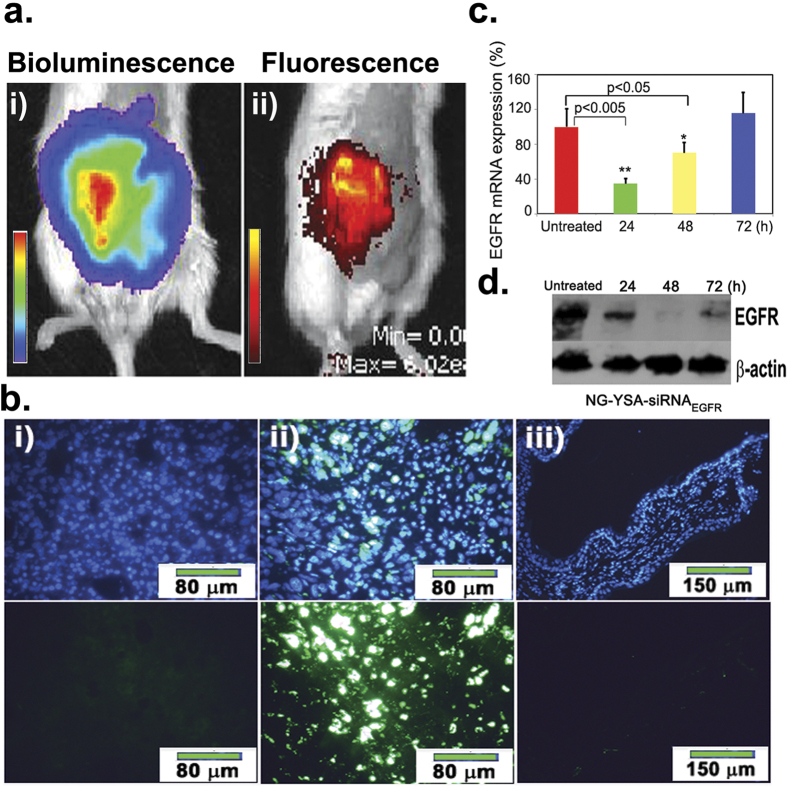
Targeted *in vivo* delivery of siRNA_EGFR_ to tumor cells significantly reduces EGFR expression. (**a)** Functionalized nanogels injected IV into tumor-bearing mice target tumor tissues *in vivo*: i) Image of the bioluminescence signal (IVIS) in tumor-bearing mice 21 days after IP injection of Hey A8-F8 cancer cells; ii) Functionalized (EphA2-targeted) nanogels IV injected with DY677-labeled negative control siRNA (2 mg siRNA/kg body weight) and imaged for fluorescence signal 6 hours after nanogel injection; (**b)** Histological evidence of targeted localization of functionalized nanogels within tumor tissues. Sections through tumor tissue from untreated (i) and functionalized nanogel injected (ii) mice were DAPI (nuclear) stained (upper panels) and scanned for florescent signal in DY677 labeled nanoparticles (lower panels). Sections through adjacent non-cancerous tissue in functionalized nanogel-injected mice (iii) display no evidence of nanogel accumulation; (**c)** qRT-PCR results demonstrate significant differences (shown are means +/−SD) between treated (NG-YSA-siRNA_EGFR_) and untreated control mice at 24h (**P < 0.005) and 48h (^★^p < 0.05) after IV injection; (**d)** Western blot analysis of EGFR protein displays reduced levels of EGFR protein at all time points relative to untreated controls but the effect is most pronounced at 48 h.

To evaluate the effectiveness of the functionalized nanogels to target tumor tissue *in vivo*, the nanoparticles were loaded with fluorescein (Dy677) labeled siRNA and injected into the tail veins (IV) of tumor bearing mice 21 days after the IP implantation of Hey A8-F8 cells. Non-invasive imaging conducted 6 hours after IV injection of nanoparticles demonstrated the presence of signal localized in the area of the tumor (Fig. 2a). Analysis of histological sections from injected mice confirmed the presence of florescent signal in the tumorous tissue but not in adjacent non-cancerous tissue (Fig. 2b).

To test the functionality of the siRNA_EGFR_ payload, nanogels carrying siRNA_EGFR_ (NG-YSA-siRNA_EGFR_, 7 mg/kg body weight-see Supplementary Fig. S5) were injected into the tail vein of tumor bearing NOD/SCID mice (18 days after the IP implantation of Hey A8-F8 cells). Cohorts of treated animals were sacrificed at 24, 48 and 72 hours post-injection and EGFR mRNA (qRT-PCR) and protein (Western blots) levels were compared between the tumorous tissues in treated and untreated mice. The results demonstrate a significant reduction in both EGFR mRNA and protein levels post-nanoparticle injection (Fig. 2c,d). Reduction in levels of EGFR mRNA were most pronounced at 24 hours and returned to untreated levels by 72 hours post-injection. In contrast, levels of EGFR protein were most significantly reduced at 48 hours post-injection and did not return to untreated control levels by 72 hours. We attribute this variability to differences in relative rates of EGFR mRNA and protein turnover^7^,^8^.

Overexpression of EGFR has been previously associated with the enhanced growth, invasion, and metastasis of a variety of human cancers^9^. The majority of epithelial cancers, including OC, have been found to display elevated levels of EGFR^10^ and elevated levels of EGFR have typically been associated with poor prognosis^11^. Despite widespread evidence for the potential clinical significance of elevated levels of EGFR in ovarian and other solid tumors, therapeutic inhibition of EGFR in cancer patients has met with limited success e.g. refs 12, 13, 14. Overall, the clinical response rates associated with EGFR inhibitors are between 10–20% across a variety of human malignancies e.g. refs 15,16 including OC e.g. ref. 17.

Despite these disappointing results, more recent *in vitro* studies suggest that siRNA-mediated knockdown of EGFR may be more effective in blocking or terminating cancer cell growth than EGFR tyrosine kinase (TK) inhibitors alone e.g. ref. 18. For example, studies carried out with prostate cancer cells have shown that EGFR, independent of its TK activity, physically associates with and stabilizes the sodium/glucose co-transporter (SGLT1) to promote glucose uptake into cancer cells thereby protecting the cells from undergoing cell death^19^. These and similar results in renal carcinomas^20^ imply that knockdown of all EGFR functions may be necessary to significantly inhibit tumor growth and that siRNA-mediated knockdown of EGFR levels may be a more effective strategy than EGFR inhibitors alone in the treatment of at least some cancers associated with elevated levels of EGFR.

**Figure 3:**
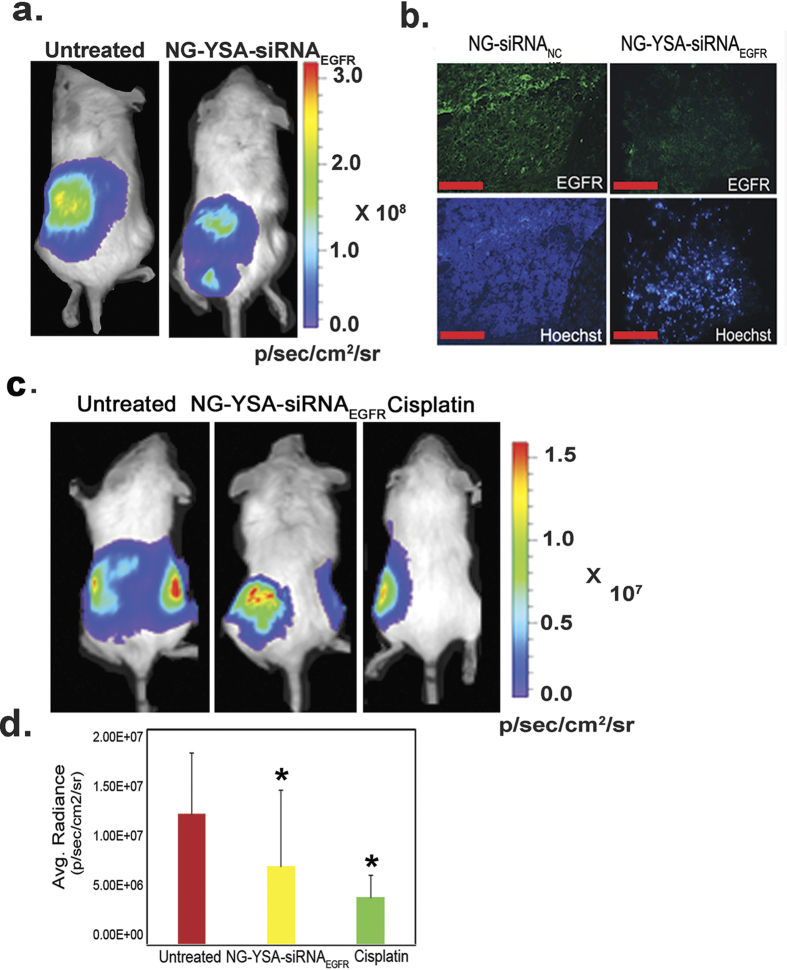
Tumor growth is reduced in tumor-bearing mice treated with NG-YSA-siRNA_EGFR._ (**a)** Bioluminescence of Hey A8-F8 tumors in a representative untreated mouse (left panel) and in a mouse 48 h after IV injection of NG-YSA-siRNA_EGFR_ nanogels (7 mg/kg body weight) (right panel); (**b**) Immunohistochemistry imaging of tumor tissue isolated from mice 48 h after IV injection of NG-YSA-NC or NG-YSA-siRNA_EGFR_ nanogels (7 mg/kg body weight) display reduced levels of EGFR protein in the NG-YSA-siRNA_EGFR_ treated mouse relative to the NG-YSA-NC treated control (EGFR-green, nuclear Hoechst staining-blue); (**c)** Mice treated with IP cisplatin (10 mg/kg body weight) display a significant reduction in tumor size relative to untreated mice and mice treated with NG-YSA-siRNA_EGFR_ after 72 h. (**d)** Region of interest (ROI) analysis was used to quantitate tumor burden in treatment groups relative to untreated controls. Bar graph displays the mean bioluminescence radiance +/−SD generated as photons/sec/cm^2^/sr (^★^p < 0.05, t-test, n = 3).

To test the effect of EGFR knockdown on OC growth in our animal model, tumor-bearing mice (18 days after the IP implantation of Hey A8-F8 cells) were injected IV with functionalized nanoparticles carrying siRNA_EGFR_ as described above. At 48 hours post-injection, tumor tissues were compared between the siRNA_EGFR_ treated and untreated mice (Fig. 3a). Knockdown of EGFR levels in siRNA_EGFR_ treated mice was confirmed by immunological staining(Fig. 3b). Tumor growth was significantly reduced in siRNA_EGFR_ treated mice relative to untreated controls (p < 0.05), although the magnitude of the effect was less than in mice treated with cisplatin alone (Fig. 3d).

The apoptosis-inducing platinum based drugs cisplatin and carboplatin have long been components of first line therapies for ovarian and other epithelial cancers^21^. A number of *in vitro* studies have linked resistance to cisplatin in ovarian and other cancer cells with overexpression of EGFR e.g. refs 22, 23, 24. However, to date, clinical trials have failed to establish a significant benefit of EGFR inhibitors in the treatment of cisplatin-resistant tumors e.g. refs 25, 26, 27, 28. More recent *in vitro* studies suggest that EGFR-mediated cisplatin resistance may be associated with EGFR’s role in glucose metabolism^29^. These findings coupled with results indicating that siRNA-mediated knockdown of EGFR levels may be a more effective strategy than TK inhibitors alone in blocking EGFR-mediated glucose metabolism^19^,^20^ suggests that a similar approach may be useful in enhancing cisplatin therapy.

Having determined that the reduction in tumor burden after treatment with siRNA_EGFR,_ while significant, was less than what is observed with treatment with cisplatin alone (Fig. 3c,d), we next tested the potential synergistic effectiveness of knockdown of EGFR when combined with cisplatin treatment. Tumor bearing mice (9 days after IP implantation of Hey A8-F8 cells) were administered a single dose of siRNA_EGFR_ bearing nanogels (NG-YSA-siRNA_EGFR_) IV as described above, followed 1 day later with administration of a single IP dose (10 mg/kg body weight) of cisplatin (Cis). Two control groups of animals were analyzed in parallel experiments: tumor bearing untreated mice and tumor bearing mice treated only with cisplatin. Tumor growth was non-invasively monitored in these mice for a period of two weeks (15 days) at which time the experiment was terminated due to the extensive size of tumor growth in untreated mice.

**Figure 4:**
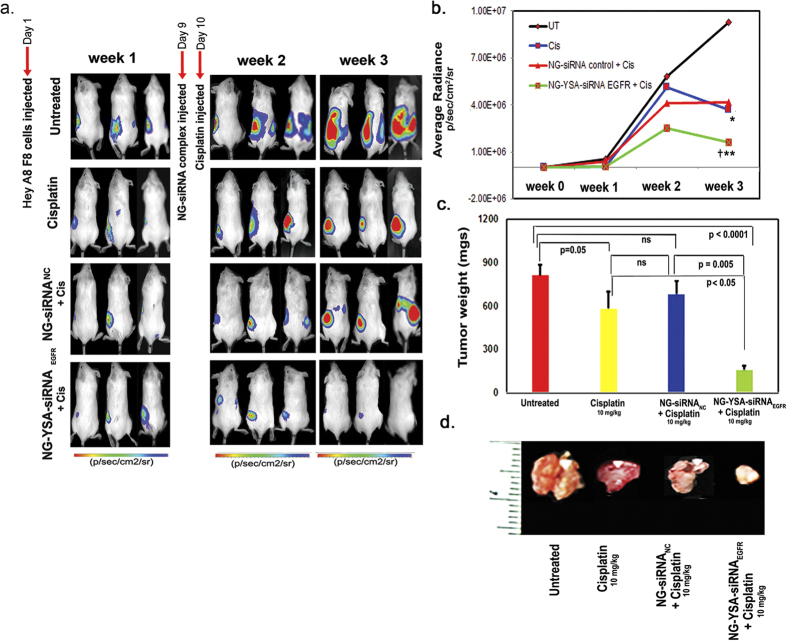
Ovarian tumor growth in untreated and treated mice. (**a)** Noninvasive bioluminescence images (radiance efficiency) of Hey A8-F8 ovarian tumor-bearing mice at weeks 1, 2 and 3 for indicated treatment groups. On day 9, tumor bearing mice were injected with a single IV dose (NG @7 mg/kg) of either NG-siRNA_NC_ (row 3) or NG-Y-siRNA _EGFR_ (row 4) followed by a single IP dose of cisplatin (10 mg/kg) on day 10; (**b)** Quantitative assessment of bioluminescence following cisplatin treatment alone or when coupled with siRNA treatments was carried out at weekly intervals. While cisplatin treatment alone resulted in a significantly reduced signal at week 3 relative to untreated controls (*p < 0.05), the combined treatment with NG-Y-siRNA_EGFR_ and cisplatin was significantly lower than cisplatin treatment alone (^†^p < 0.01) and the most significantly reduced signal relative to untreated controls (**p < 0.01); (**c)** Consistent with the bioluminescence assays, tumor mass was most significantly reduced in mice exposed to the combined NG-Y-siRNA _EGFR_ and cisplatin treatment. Bar graphs display average tumor weight ± standard deviation; (**d)** Dissected tumors from representative mice after 3 weeks of treatment.

As shown in Fig. 4, the responses of cisplatin treated control mice displayed a significant reduction in tumor growth relative to untreated mice (p < 0.05). This confirms the well-established effectiveness of cisplatin therapy on OC growth. In contrast, the cisplatin treated mice injected with nanoparticles carrying EGFR siRNA displayed a significantly greater reduction in tumor growth relative to controls. These findings are consistent with the hypothesis that knockdown of EGFR can significantly enhance the therapeutic benefits of platinum based drugs on OC growth *in vivo* (see also Supplementary Fig. S6).

The effectiveness of targeted knockdown of gene expression by siRNA has long been established *in vitro* but has not yet seen widespread clinical application due to a lack of effective ways to deliver siRNA *in vivo*. This limitation is rapidly fading with the recent development of a number of promising strategies for the effective delivery of siRNAs and other potentially therapeutic RNAs in animals^30^. Within the context of cancer biology, these breakthroughs open the possibility of directly testing *in vivo* a variety of novel strategies for cancer treatment suggested by extensive studies conducted in cancer cell lines.

In this study, we developed and utilized a functionalized nanohydrogel siRNA delivery system and a mouse model of OC to test predictions from cell line studies that knockdown of EGFR may be of clinical significance in the treatment of epithelial tumors especially with respect to the enhancement of platinum based therapies. Our results support these predictions and suggest that targeted delivery of EGFR siRNA may be an effective strategy for the treatment of ovarian and other epithelial tumors associated with elevated levels of EGFR and especially those demonstrating resistance to platinum-based therapies.

## Methods

### Construction and characterization of Hey A8-F8 cells

Hey A8 cells (provided by Dr. Gordon B. Mills, MD Anderson Cancer Center, Houston, TX) were maintained in RPMI 1640 (Mediatech) supplemented with 10% FBS (Fetal Bovine Serum; Atlanta Biologicals) and 1% antibiotic-antimycotic solution (Mediatech). Adherent monolayer cultures were maintained at 37 °C in 5% CO_2_. All reagents were purchased from Sigma-Aldrich (St Louis) and used as received unless otherwise noted.

To establish stable constitutively expressing luciferase transfectants, Hey A8 cells were transfected with the pGL4.51 Luciferase Reporter Vector (Promega) including the neo resistant gene using Lipofectamine 2000 transfection reagent (Invitrogen). Transfected cells were selected for antibiotic resistance to geneticin (G418 sulphate, GIBCO) that serves as a selectable marker for stably transfected cells. After selection, multiple sub-clones of Hey A8 cells stably transfected with the gene encoding firefly (*Photinus pyralis*) luciferase were assessed using the Bright-Glo™ Luciferase Assay System (Promega) according to the manufacturer’s instructions. A stable Hey A8 clone expressing high levels of luciferase (Hey A8-F8) was isolated and maintained in standard culture media (see below) with 50 μg/mL G418 (Invitrogen).

Quantitation of firefly luciferase expression in Hey A8-F8 cells was carried out by directly adding a D-luciferin substrate at a final concentration of 150 μg/ml, (PerkinElmer) into cells plated in a 96-well plate. Bioluminescence imaging (BLI) was measured by using IVIS (*in vivo* imaging system) Lumina II system (PerkinElmer) 5 minutes after addition of the substrate, which demonstrated a highly significant correlation (R^2^ = 0.99) between total photon flux and number of Hey A8-F8 cells present *in vitro*.

### Nanohydrogel synthesis

Nanohydrogels were synthesized *via* emulsion precipitation polymerization as previously described^31^. In a typical core nanogel synthesis, 0.772 g N-iso-propylmethacrylamide (NIPMAm) monomer, 0.0.0191 g N, N’-methelene-bis (acrylamide) (BIS) cross linker and 0.1135 g sodium dodecyl sulfate (SDS) surfactant were dissolved in 49 ml water. The solution was stirred under nitrogen. After the solution was brought to 70 °C under nitrogen, 0.1 mM acrylamido-fluorescein (AFA) co-monomer, and 0.5 ml of a 0.01 g/ml aqueous ammonium persulfate (APS) solution were added to initiate polymerization. The polymerization was carried out at 70 °C for 4 hours under nitrogen. The core nanogels were used as seed for the addition of a shell layer. The detailed procedure of the shell synthesis has been reported previously^31^. Briefly, a 50 mM monomer solution with molar ratios of 97.5% NIPMAm, 2% BIS, and 0.5% aminopropyl methacrylate (APMA, Poly sciences) is prepared in 39.5 mL of dH_2_O. When the temperature stabilized at 70 °C, the reaction is initiated by adding 0.5 mL aliquot of 0.05 M APS. The reaction proceeds for 4 hours under N_2_ gas. After cooling down to room temperature, core-shell particles are filtered through a 0.2 um filter and the size of the core-shell nanogel is measured by dynamic light scattering (DLS) equipment (Zetasizer Nano-ZS S-90).

### Nanohydrogel functionalization with ephrin mimetic, YSA peptide

The YSA (YSAYPDSVPMMS) peptide^32^ mimics the ligand ephrin-A1, and binds to the ephrin type-A2 (EphA2) receptor. This 12-mer amino acid sequence (GenScript) was conjugated to the nanohydrogels via maleimide coupling to the cysteine residue on the C-terminal end of the peptide as previously described^31^. Briefly, a solution of ethyl-3-dimethyl amino propyl-carbodiimide hydrochloride (EDC, Pierce, Carlsbad, CA) and N-hydroxysulfosuccinimide (sulfo-NHS, Thermo Fisher Scientific) was added to maleimidocaproic acid in pH 6.0 MES buffer to activate the acid groups for 30 minutes at room temperature. This solution was then added to particles and reacted for 2 hours on a shaker table. The particles were centrifuged to remove unreacted material, and peptide added to the particles and reacted overnight. Particles were again centrifuged to remove unreacted materials described above and subjected to lyophilization for 72 hours.

### Loading of nanohydrogels with siRNA

The nanohydrogels are loaded with siRNA using the “breathing in” method as previously described^31^. Briefly, 4 mg of lyophilized nanogel was rehydrated in 250 μl of siRNA EGFR (Life Technologies Corporation, Cat # 4390825) or non-targeting siRNA negative control (Life Technologies Corporation Cat # 4390843) (20 μM stock made in sterile PBS) for 12 hours with rotation at 4 °C. After the siRNA was encapsulated in the nanogels, they are centrifuged and re-suspended to a final concentration of 10 mg/mL in reduced serum media (OPTI-MEM, Thermo Fisher Scientific). The final concentration of siRNA is typically 16–17 μg siRNA/mg of nanogels.

### Introduction of nanoparticles to cells *in vitro*

Hey A8-F8 cells were plated at a density of ~2 × 10^4^ cells per well in a 24-well plate in 500 μL RPMI 1640 with 10% FBS. Nanoparticles carrying EGFR siRNA (NG-YSA-siRNA_**EGFR**_) were added drop-wise to each well. The final siRNA concentration in each well was 125 nM. Following a 4–6 hour incubation period, the culture media was replaced by pre-warmed cultured media with 10% FBS and allowed to incubate for another 48–72 hours. *In vitro* gene silencing transfection efficiency is established by using quantitative RT-PCR and immunofluorescence methods.

### RNA extraction and quantitation

Total RNA was extracted from Hey A8-F8 cells or tumor tissue using the RNeasy mini kit (Qiagen). RNA concentration was measured using a Nano Drop 1000 Spectrophotometer V3.2 (Nano Drop). RNA samples were converted into first-strand cDNA with the Superscript III First-strand Synthesis System (Invitrogen) using 500 ng to 1 mg of RNA. Real time PCR for EGFR was performed using the TaqMan gene expression assay according to the manufacturer’s protocol on a Prism 7500 TaqMan qPCR machine (Applied Biosystems) using β-actin as internal control. Relative mRNA expression was calculated using the comparative threshold cycle (Ct) method.

### Immunofluorescence

Cells were placed on 8-well chamber slides (Nunc™ Lab-Tek™ Chamber Slide, Thermo Fisher Scientific) and allowed to adhere overnight. After fixation in 4% paraformaldehyde in PBS, cells were permeabilized in 0.2% Triton X-100 in PBS for 15 minutes, and then blocked with 3% goat serum in PBS for 1 hour. Cells were incubated with the primary antibody diluted in blocking buffer at room temperature for 2 hour (EGFR antibody Cat # sc-120, 1:50; EphA2 antibody Cat # sc-924, 1:50; Santa Cruz) followed by a 30 minutes incubation with appropriate secondary antibody (Cat # A11001, #A 1:1,000; Invitrogen). After washing with PBS, cells were counter stained with 4,6-diamidino-2-phenylindole (DAPI, 1:1,000) (Thermo Fisher Scientific) for nuclear stain. The slides were examined under a fluorescence microscope (Nikon E600 microscope).

### Mouse model

Five to six**-**week-old female severe combined immunodeficiency (SCID) mice (NOD.CB17-Prkd^scid^/NcrCrl, strain code 394) were purchased from Charles River Laboratories. The mice were housed and maintained under pathogen-free conditions in facilities approved by the American Association for Accreditation of Laboratory Animal Care and in accordance with current regulations and standards of the U.S. Department of Agriculture, U.S. Department of Health and Human Services, and NIH. All studies were approved by the Georgia Institute of Technology Institutional Animal Care and Use Committee.

To generate the mouse model, Hey A8-F8 cells (5 × 10^6^ cells/0.5 mL RPMI 1640 with antibiotics) were injected intraperitoneally into the mice. Prior to *in vivo* injections, the cultured cells were trypsinized and centrifuged at 800 rpm for 5 minutes at room temperature, washed and reconstituted in serum-free RPMI 1640 with antibiotics (Life Technologies/ Invitrogen). About 5 × 10^6^ living cells were injected intraperitoneally to the NOD/SCID mice and the luciferase bioluminescence imaging was used to monitor tumor growth over time.

### Bioluminescence imaging of tumor growth

Mice were anesthetized with ketamine/xylazine/acepromazine and subsequently injected intraperitoneally with luciferin potassium salt in PBS at a dose of 150 mg/kg (0.05 mL/10 g of body weight). After 10 minutes, the animals were placed in the imaging chamber and imaged with a cooled charge-coupled device camera. Dorsal and ventral bioluminescence imaging was acquired using an IVIS Spectrum CT *In Vivo* Imaging System (Perkin Elmer Inc.) at the optimal imaging time. Living Image 4.4^TM^ software (PerkinElmer Inc.) was used to compute regions of interest (ROI) and integrate the total bioluminescence signal in each ROI. Signal intensity was quantified either using bioluminescent (BL) average radiance measurements (photons/sec/cm2/sr) or total flux (photons/sec).

### Fluorescence imaging of *in vivo* delivery of siRNA by nanogel

Negative control siRNA (NC: 5′-UAGCGACUAAACACAUCAAUU-3′) labeled with DY677 and purified by HPLC for *in vivo* use was procured from ThermoFisher Scientific (https://www.thermofisher.com/order/custom-genomic-products/tools/sirna/). Tumor (IP) bearing mice were treated with single IV injection of EphA2-targeted nanogel (NG-YSA-siRNA_NC_) loaded with DY677-labeled non-targeting siRNA (2 mg/kg body weight of siRNA) and imaged 6 hours post injection for DY677 fluorescence and bioluminescence using the IVIS Lumina II system under inhalational anesthesia. Delivery of EphA2-particles into tumor tissue after IV administration of 1 μg/kg NG-YSA-SIRNA_EGFR_ was confirmed by epifluorescence microscopy of DAPI-stained tumor tissue sections using DAPI and FITC filter settings for Zeiss LSM 700. Nanogels containing 4-acrylamidofluorescein, which was added as a co-monomer during their synthesis, were detected as green fluorescent particles.

### Histological analysis

For histological evaluation, excised tumors were embedded in optimal cutting temperature (OCT) solution without fixing, and frozen using liquid nitrogen solution. Thin cryo sections (7 μm thickness) were collected on glass slides, and fixed in cold acetone for 10 minutes, stored at −80 °C. Stored sections were removed from the −80 °C freezer and allowed to reach room temperature 30 minutes before histology. After PBS washes, slides were blocked with 3% normal goat serum in PBS for 30 minutes at room temperature followed by incubation with primary antibody (anti-EGFR, 1:500 dilution, # sc-120) in blocking solution overnight at 4 °C or 1 h at 37 °C in a humid chamber. After two PBS washes (5 minutes each), the tissue sections were incubated with Alexa Fluor 488 dye (green, Invitrogen, dilution 1:500) labeled secondary antibodies that was used to detect EGFR positive cells. After an additional wash with PBS, the tissue sections were incubated with anti-Ki67 polyclonal antibody (# sc-15402, Santa Cruz Biotechnologies) overnight at 4 °C. Ki67 is a well-known biomarker of proliferative growth and can be used a proliferative index in quantitative image analysis^33^.

After a brief wash with PBS, the tissue sections were counter stained with Hoechst 33342 for visualization of nuclei. Images were taken using fluorescence microscopy (Nikon E600 microscope). All tumor tissues were randomly cut into 6–8 histological sections and stained with hematoxylin and eosin (H&E).

### Western blots

Tumor tissues were excised and snap frozen in liquid nitrogen and stored at −80 °C. A portion of frozen tumor was lysed in ice cold lysis buffer (50 mM Tris- HCl, pH 7.5, 150 mM NaCl, 2 mM EDTA, 2 mM EGTA, 1 mM sodium orthovanadate, 2.5 mM sodium pyrophosphate, 1 mM -gycerolphosphate, 1 mM phenylmethanesulfonyl fluoride, 10 μg/ml aprotinin, 10 μg/ ml leupeptin, 1% Triton X- 100, and 5% glycerol and the lysates homogenized with a mortar and pestle on ice, centrifuged at 14 000 rpm for 15 minutes at 4 °C, and the supernatant was stored at −80 °C.

The total protein concentration of the supernatant was determined using a protein assay reagent kit (Bio-Rad Laboratories, Inc.). To the lysates, equal volumes of 2X Laemmli sample buffer were added and the samples were heated to 90 °C for 5 minutes. Equal amounts of proteins were separated by 4–20% gradient precast TGX gel (Bio-Rad Laboratories, Inc.) and transferred to nitrocellulose membrane (Bio-Rad Laboratories, Inc.). Membranes were blocked with 5% nonfat dry milk in 10 mM Tris buffered saline, pH 7.5 plus 1% Tween 20. After blocking, the membranes were probed with the primary antibody epidermal growth factor receptor (EGFR antibody Cat # sc-120, 1:500 dilution, Santa Cruz Biotechnology) for overnight at 4 °C with gentle rocking. Appropriate secondary antibody was used at 1:5,000 dilutions. After incubation with goat anti-mouse horseradish peroxidase–(HRP) conjugated secondary antibody (sc-2005, Santa Cruz). EGFR protein was visualized using the enhanced chemiluminescence detection system (Pierce). Equal loading was confirmed by detection of β-actin (Cat# Mab-1501, 1:5,000 dilution, Millipore).

## Additional Information

**How to cite this article**: Satpathy, M. *et al.* Targeted *in vivo* delivery of EGFR siRNA inhibits ovarian cancer growth and enhances drug sensitivity. *Sci. Rep.* **6**, 36518; doi: 10.1038/srep36518 (2016).

**Publisher’s note:** Springer Nature remains neutral with regard to jurisdictional claims in published maps and institutional affiliations.

## Supplementary Material

Supplementary Information
