# Supplementary material for: Targeted in vivo delivery of EGFR siRNA inhibits ovarian cancer growth and enhances drug sensitivity
Source: Sci Rep. 2016 Nov 7;6:36518. doi: 10.1038/srep36518 (PMC5098171; doi:10.1038/srep36518)
Supplement: Supplementary Information [file srep36518-s1.pdf]

Targeted *in vivo* delivery of EGFR siRNA inhibits ovarian cancer growth and enhances drug sensitivity (Minati Satpathy, Roman Mezencev, Lijuan Wang, and John F. McDonald)

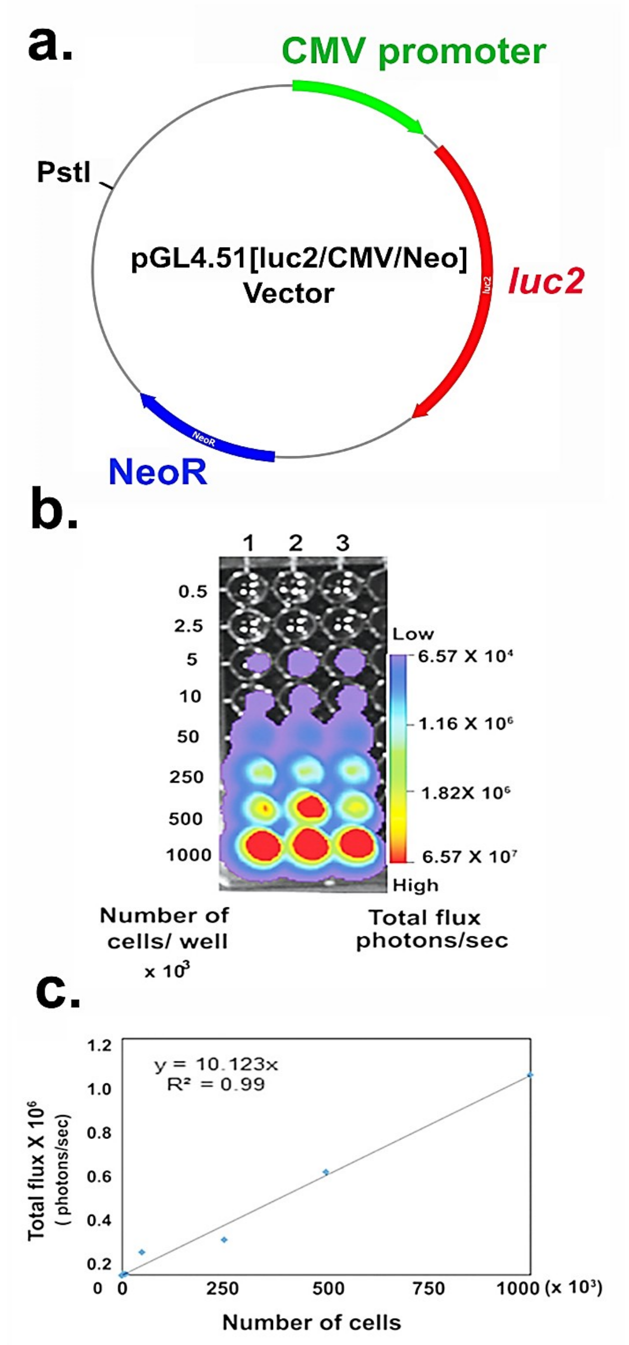

Supplementary Figure S1. Generation and characterization of stable luciferase positive ovarian cancer cell line (Hey A8-F8). a) Schematic representation of the pGL4.51 luciferase reporter vector with a CMV enhancer/ promoter, firefly luciferase gene (*luc2*) and mammalian selective marker gene for neomycin resistance used for transforming the Hey A8 cells with Lipofectamine 2000; **b)** Bioluminescence signal of serially diluted transformed Hey A8-F8 luciferase positive ovarian cancer cells was measured by *in vivo* imaging; **c)** Correlation plot of total photon flux vs. number of plated cells (R^2 =0.99) demonstrating a linear relationship between number of cells and the total photon flux.

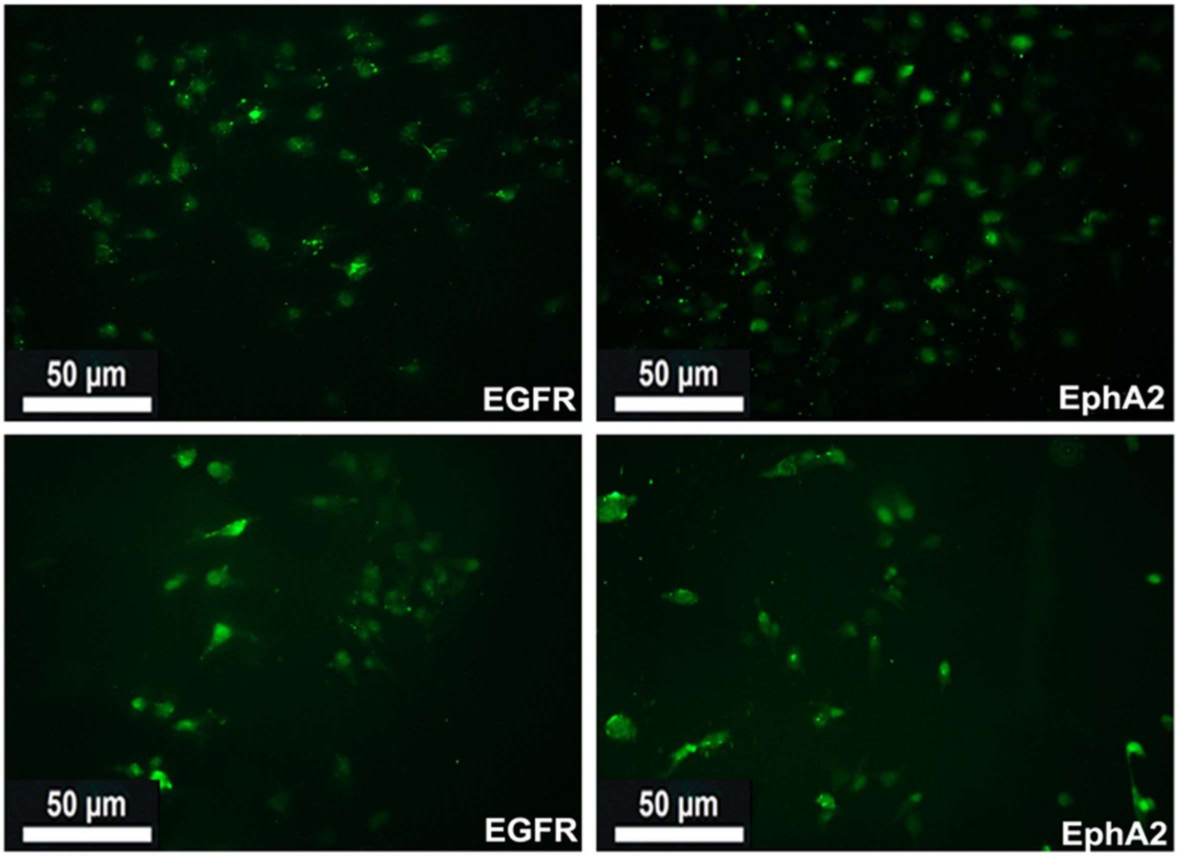

Supplementary Figure S2. EGFR and EphA2 expression levels in Hey and Hey A8-F8 ovarian cancer cells. Representative images showing immunofluorescence staining for EGFR (left) and EphA2 (right) proteins (green) in Hey (Upper panel) and Hey A8-F8 (Lower panel) ovarian cancer cell lines. Magnification: 20x

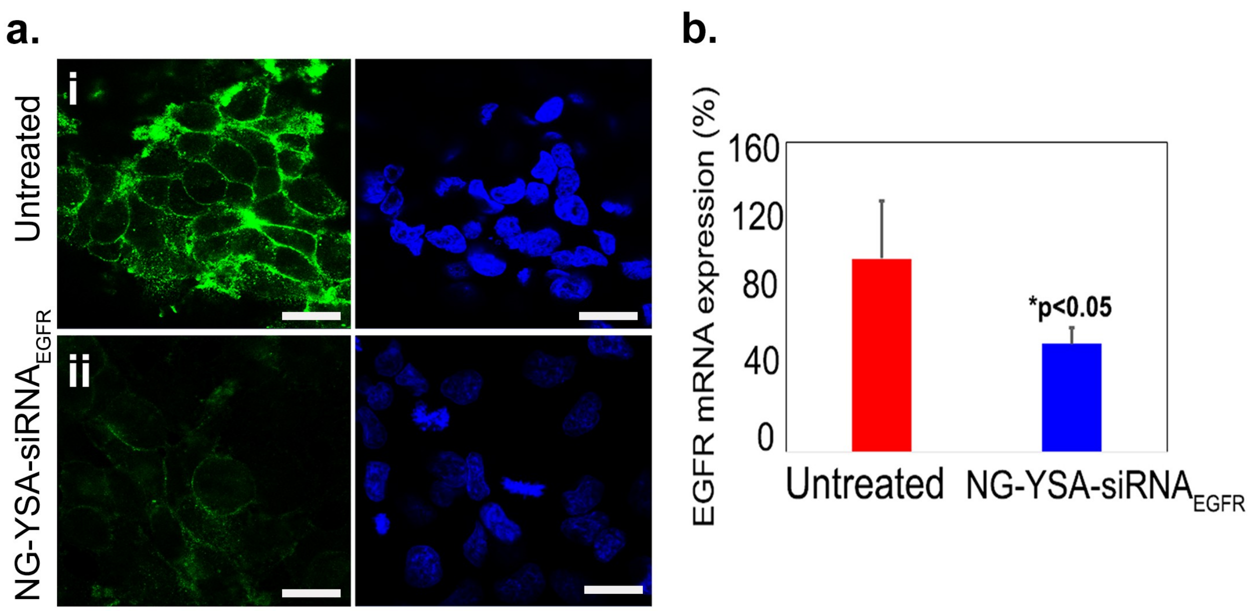

Supplementary Figure S3. Gene silencing in Hey A8-F8 ovarian cancer cells (*in vitro)* mediated by NG-YSA-siRNA$_{EGFR}$. Cells were transfected with nanogels carrying EGFR siRNA (NG-YSA-siRNA$_{EGFR}$) and EGFR expression was monitored 48 hours post-transfection. **a)** Representative immunofluorescence imaging displaying reduction in EGFR levels (green color) in cells transfected with NG-YSA-siRNA$_{EGFR}$ nanogels (ii) relative to the untreated control cells (i). Cells were counter stained with DAPI (blue). Scale bar-100 ∝m **b.** RNA was extracted from control and treated cells and EGFR expression measured by qRT-PCR. Shown are the averages of 3 replicates +/- SD (*p<0.05).

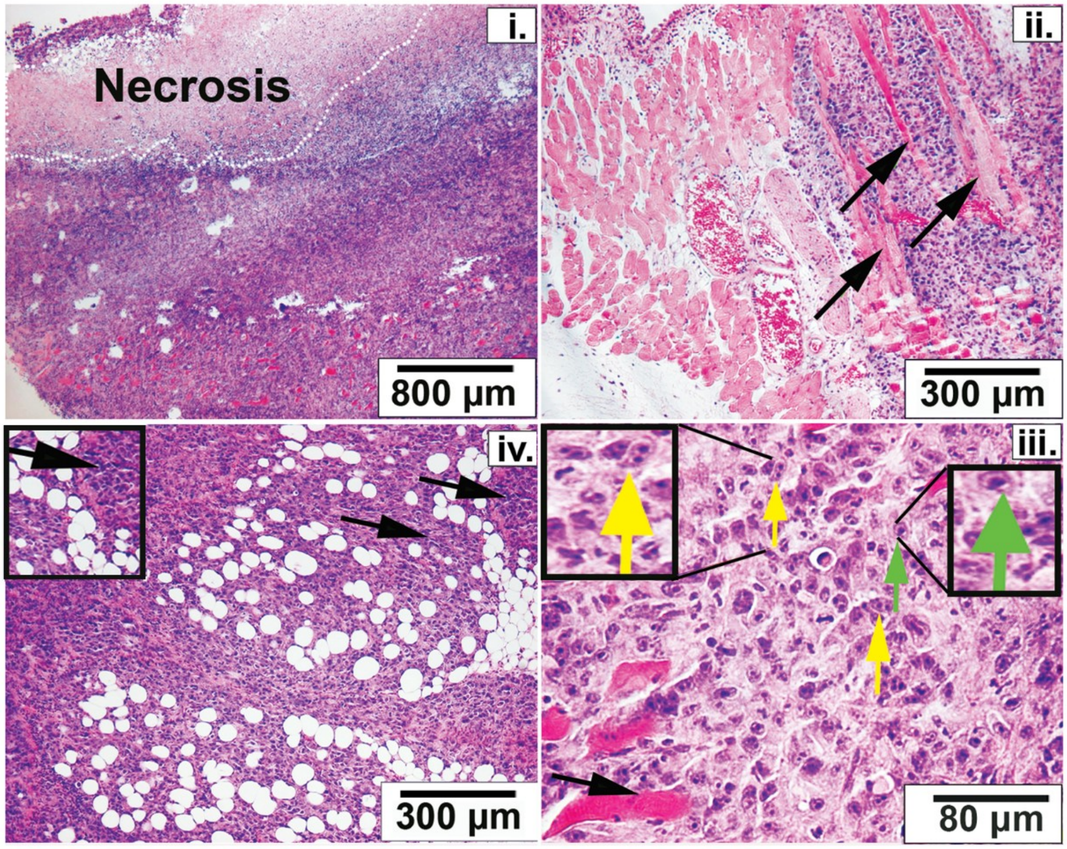

Supplementary Figure S4. Histopathological analysis of the intraperitoneal tumors. Stained tissue sections displayed solid growth pattern with extensive necrotic areas (i); muscle (ii and iii) and adipose (iv) tissues invaded by more deeply stained tumor cells (black arrows). Pleomorphic nuclei (yellow arrows) and distinct nucleoli with irregular chromatin (green arrows) are apparent in zoomed images inside the black rectangular box. Overall histology is consistent with high-grade serous ovarian carcinoma. Hematoxylin and eosin (H & E) staining; Magnification: (i) 40x, (ii and iii) 100x, (iv) 400x.

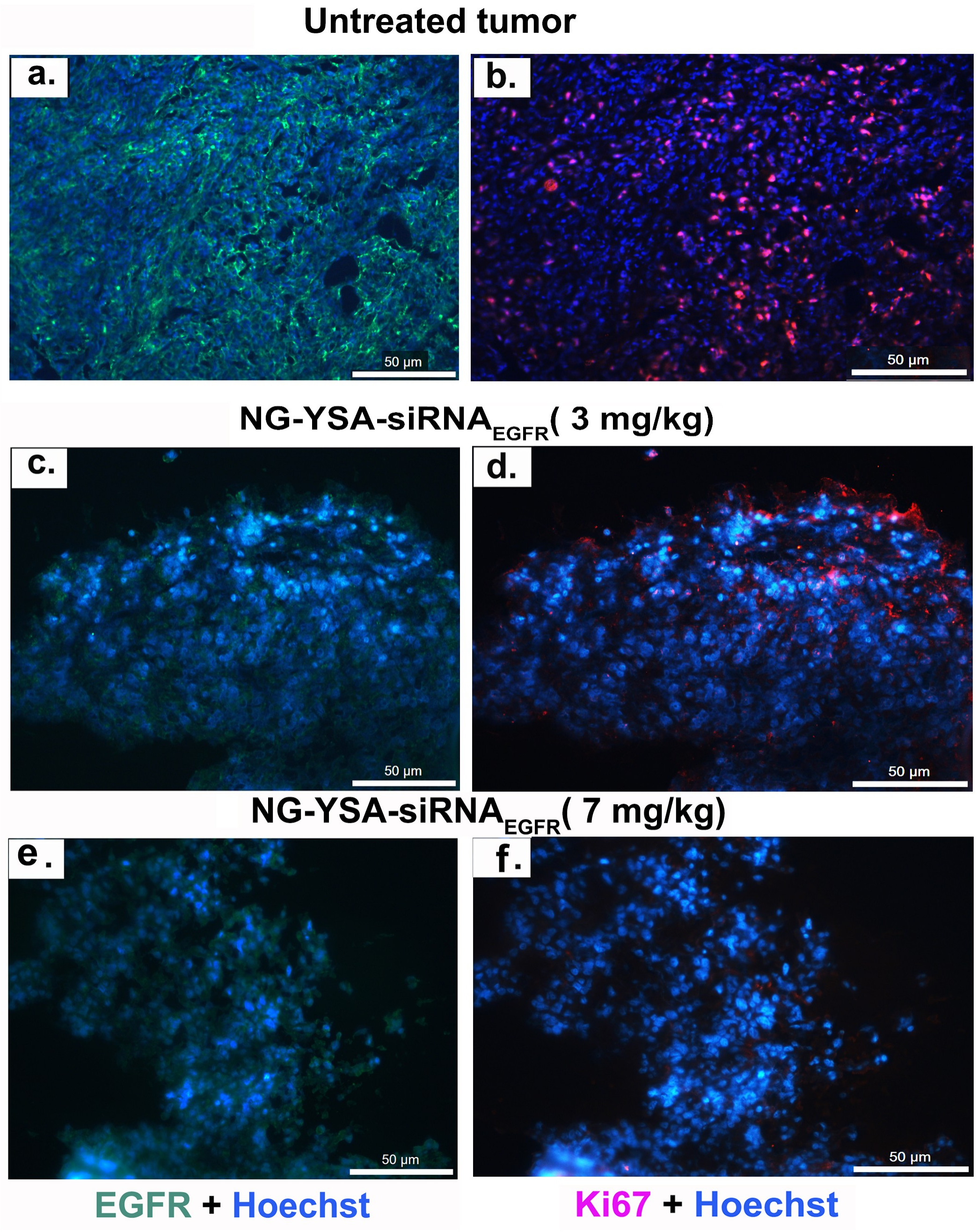

Supplementary Figure S5. Determination of dosage level of NG-YSA-siRNA$_{EGFR}$ nanogels required to block proliferation of ovarian cancer cells *in* vivo. Two concentrations (3 mg/kg body weight or 7 mg/kg body weight) of siRNA-EGFR loaded nanogels (NG-YSA-siRNA$_{EGFR}$) were injected into the tail veins (IV) of tumor bearing mice 18 days after the IP implantation of Hey A8-F8 cells. 48 hours after treatment, mice were sacrificed and tumors removed for histological analysis. The results of immunofluorescence imaging of EGFR and Ki67 (a biomarker of proliferative growth) expression levels in treated mice and untreated controls demonstrate that while EGFR (green, Fig 5a) levels are significantly reduced after treatment with a minimum of 3 mg/kg body weight of nanogels ($p>0.001$) (c, e), a dosage of 7 mg/kg body weight is needed to knock out levels of Ki67 ($p>0.005$) (f). Nuclei were counterstained with Hoechst 33342 (blue).

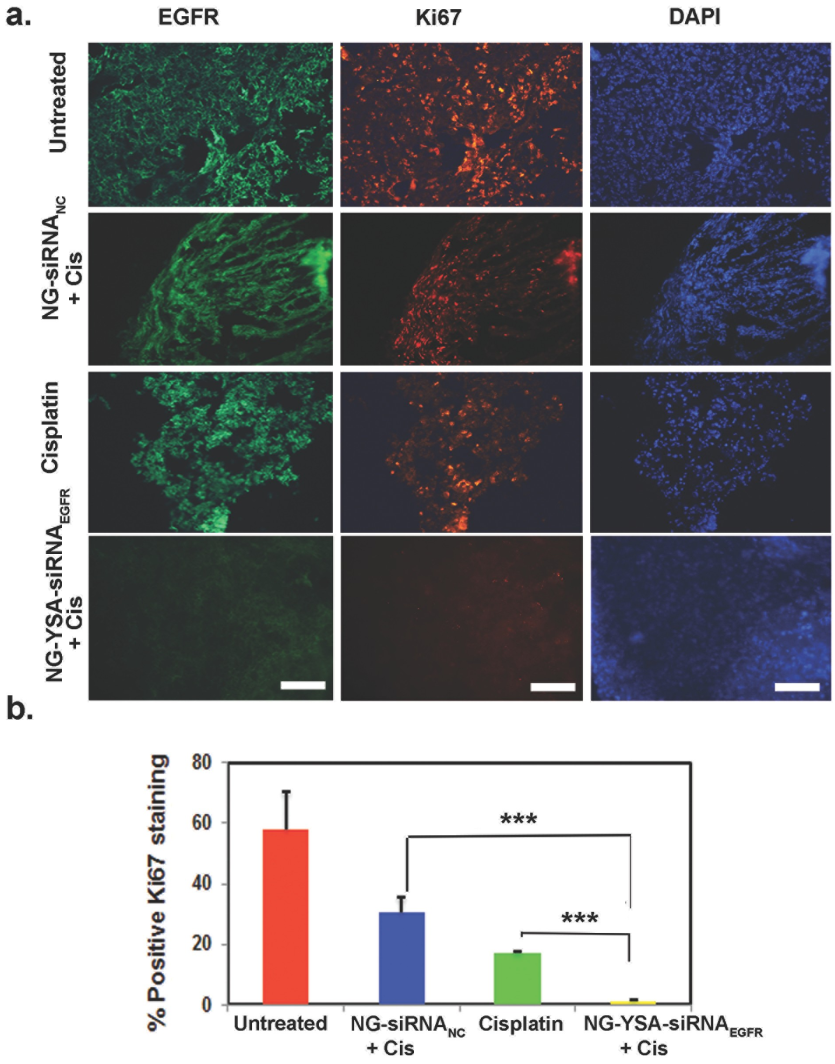

Supplementary Figure S6. Reduced levels of EGFR expression and tumor proliferation are correlated in treated mice. a) Immunohistochemistry imaging of EGFR and Ki67 (a biomarker of proliferative growth) expression levels in tumor sections from representative treated mice and untreated controls. Nuclei were counterstained with DAPI (blue) to display total cell count per section; b) A bar graph showing the quantitative analysis of Ki67-positive cells in the indicated groups from 3 different samples. (***$p < 0.005$)
